# PReS-FINAL-2226: Assessment of autonomic functions in children with Familial Mediterranean Fever by using heart rate variability measurements

**DOI:** 10.1186/1546-0096-11-S2-P216

**Published:** 2013-12-05

**Authors:** K Fidanci, M Gulgun, A Kilic, C Acikel, E Demirkaya, F Gok, S Ozen

**Affiliations:** 1 Gulhane Military Medical Academy, Ankara, Turkey; 2 Hacettepe University, Ankara, Turkey

## Introduction

Familial Mediterranean Fever (FMF) is an autoinflammatory disorder characterized by recurrent fever associated with inflammation of serous membranes. There is no study reporting the assessment of autonomic functions by using heart rate variability (HRV) in children with FMF. HRV is a practical and reliable method for evaluation of autonomic functions. HRV studies have pointed to the presence of autonomic dysfunctions in many autoinflammatory disorders, possible contributing factors to ventricular tachyarrhythmias and sudden cardiac death in these patients.

## Objectives

In this study, we investigated possible alterations in cardiac autonomic functions and other probable cardiac effects in children with FMF by HRV analyses and conventional echocardiography.

## Methods

The study population included 70 consecutive patients with FMF who were examined at our Cardiology and Rheumatology Departments (27 female; mean age 11.14 ± 3.536 years) and 50 healthy control subjects (20 female; mean age 10.68 ± 3.107 years). All FMF patients enrolled in our study fulfilled the clinical criteria for FMF. The control group was consisted of fifty healthy volunteers matched for age and gender without history of any cardiac or systemic inflammatory disease. All patients were using regular colchicine therapy, clinical and laboratory assessment of FMF patients were performed during an attack-free period.

In each patient, it was performed twelve lead electrocardiography (ECG) at 25 mm/s (paper speed), 24 h ambulatory electrocardiographic monitorization (AECG), and transthoracic echocardiography by a Siemens Acuson Sequoia C256 cardiac ultrasonographic scanner, with 2.5- to 3.5-MHz transducers.

## Results

It was noted that SDNN (standard deviation of all NN intervals) value was lower in patients with FMF as compared to the control group. Frequency-dependent HRV parameters were similar in both groups. There was no difference in patient and control groups in terms of conventional echocardiographic parameters.

## Conclusion

Studies with larger cohorts and more comprehensive methods are required to assess the presence and consequences of possible autonomic dysfunction in children with FMF.

## Disclosure of interest

None declared.

